# Physical partner violence, women’s economic status and help-seeking behaviour in Dar es Salaam and Mbeya, Tanzania

**DOI:** 10.1080/16549716.2017.1290426

**Published:** 2017-05-09

**Authors:** Seema Vyas, Jessie Mbwambo

**Affiliations:** ^a^ Department of Epidemiology and Biostatistics, Kilimanjaro Christian Medical University College, Moshi, Tanzania; ^b^ Department of Psychiatry and Mental Health, Muhimbili University of Health and Allied Sciences, Dar es Salaam, Tanzania

**Keywords:** Tanzania, physical partner violence, help-seeking, women’s economic status

## Abstract

**Background**: Women’s responses to partner violence are influenced by a complex constellation of factors including: psychological attachment to the partner; context of the abuse; and structural factors, all of which shape available options for women outside of the relationship.

**Objective**: To describe women’s responses to physical partner violence; and to understand the role of women’s economic resources on their responses.

**Methods**: Cross-sectional data from Dar es Salaam and Mbeya, Tanzania. Multivariate logistic regression was used to explore the relationship between women’s economic resources and their responses to violence.

**Results**: In both sites, among physically abused women, over one-half experienced severe violence; approximately two-thirds had disclosed the violence; and approximately 40% had sought help. Abused women were more likely to have sought help from health services, the police and religious leaders in Dar es Salaam, and from local leaders in Mbeya. Economic resources did not facilitate women’s ability to leave violent partners in Dar es Salaam. In Mbeya, women who jointly owned capital assets were less likely to have left. In both sites, women’s sole ownership of capital assets facilitated help-seeking.

**Conclusion**: Although support services are being scaled-up in Tanzania, efforts are needed to increase the acceptability of accessing such services.

## Background

Violence by an intimate partner is one of the most common forms of violence against women and the extent of the problem is vast. Early population-based surveys suggested that globally between 10% and 67% of ever-partnered women have been physically assaulted by an intimate partner at some time in their lives [1]. The World Health Organization’s (WHO) multi-country study on women’s health and domestic violence against women (WHO study) – a 15-site, 10-country, population-based survey conducted in Africa, Asia, Europe and Latin America – found that between 15% and 71% of ever-partnered women had been physically and/or sexually assaulted by a male partner since the age of 15 [2].

In addition to direct injury or loss of life, partner violence increases women’s vulnerability to a range of serious physical, mental and sexual and reproductive health consequences [3–5]. Despite these negative consequences, many women continue to remain in abusive relationships, often keeping their experiences of violence hidden. The WHO study documented that among women who had ever been physically abused by a male partner, between 21% (in Namibia) and 66% (in Bangladesh) had not disclosed the violence to anyone, and that among those who had disclosed, women were most likely to have told their family or friends and the majority in all sites had never sought help from formal services [6].

### Why women stay in abusive relationships

Theories why women stay in abusive relationships initially focussed on psychological reasons with scholars from the US observing that some women had developed an emotional bond or attachment with their abusers meaning that they were less likely to leave [7–9]. Another feature that influences women’s responses to partner violence is how women view the circumstance of the abuse that they experience. For example, empirical evidence from population-based studies has found that women who experience more severe and more frequent violence, or who fear some sort of irreparable damage either to themselves or to their children, are more likely to seek intervention or to, at least temporarily, separate [10–14].

A third area of research has focussed on how structural factors – e.g. unequal power relations, availability of response services and societal norms – that determine women’s available options outside of the relationship, shape women’s responses to abuse. The fewer economic resources (employment, education or assets) a woman has or the more economically dependent a woman is on her partner, the fewer alternatives she has outside of the relationship, and the more likely she is to tolerate abuse and the less likely she is to disclose the violence, seek help or leave an abusive relationship [13,15–17]. Identified as key to greater empowerment or bargaining power, employment not only provides financial independence, it also provides women with a support network [18–20]. Studies in the US have found that abused women who were employed were more likely to initiate divorce proceedings, go to the police or leave the relationship [9,13,21]. The emphasis on employment as the route out of abuse is, however, argued to be simplistic and culture and context are said to determine women’s ability to act on their behalf [22–26]. Instead, studies in low- and middle-income countries (LMIC) have found education, rather than employment, to be the empowering resource enabling women to separate [11,14,25]. The reason for this could be that higher-educated women are less likely to tolerate violence or that they are able to establish greater networks such as links to local organisations [11,25].

Meeting the practical needs of women, by e.g. shelters/safe spaces, housing, financial resources, medical advice and physical and emotional safety, is essential for women to remain separated [21,27,28]. Women want help and the more support services that are available to them the more likely they are to seek help and the less likely they are to return. Alternatively, while some women use services to intentionally leave, others use it as a ‘bluff’, that is, the woman has no intention to permanently separate but uses available support services to signal an intention of doing so in order to reduce or prevent future episodes of violence [29]. This signalling of intent, however, may be a limited option for women in LMIC if multi-sector response services for abused women are inadequate.

Finally, societies where power imbalances between men and women mean that men are dominant over women and violence between spouses is considered normative may also play a significant factor in women’s response to abuse. Social pressures that place the burden of family harmony on women prevent them from seeking help because they fear either stigma, that they will not be believed or that they will jeopardise family honour [14,30–32].

The responses to intimate partner violence that women adopt are, therefore, complex and influenced by an interrelationship of factors. The aim of this study is to understand the role of women’s economic status on their responses to partner violence in Dar es Salaam (DSM) and Mbeya, Tanzania. Specifically, this study seeks to describe women’s responses to partner violence; to understand whether women with higher educational attainment, or who earn money, or who own capital assets are more likely to seek help and/or separate from their violent partner; and to assess whether women’s responses differ by the two study sites.

#### Research settings

DSM is Tanzania’s commercial centre with a high concentration of trade and services compared to other parts of Tanzania. The population is ethnically mixed and figures from the latest (2012) census document that 4.4 million people live in the city [33]. By contrast, Mbeya is a more provincial area in the southwest of the country with a population of 2.7 million (in 2012) of which two-thirds is rural [33].

The majority of Tanzania’s adult population are economically active with a labour force participation rate that has remained consistently high (approximately 90% over the last two decades).^1^ ^1^
http://databank.worldbank.org/data/home.aspx (accessed 24 October 2016). The dominant employment sector is agriculture where, according to the 2006 Integrated Labour Force Survey (ILFS), 79% of economically active women and 70% of men work [34]. The employment rate in DSM was 77% among males and 59% among females and almost 60% of households had one member engaged in informal sector work in 2006 [34]. The most common employment industries for both men and women were trading and agriculture followed by transport, manufacturing and construction (for men) and hospitality and private home-based enterprises (for women). While employment characteristics for Mbeya Region are not published in the ILFS, using ‘Other urban’ as a proxy for this setting yields over half (54%) of households as being engaged in informal-sector activities and a very high employment rate – 85% male and 78% female [34].

### Physical partner violence against women in Tanzania

Prevalence of physical partner violence against women in Tanzania is high. The WHO study documented that 33% of ever-partnered women in DSM and 47% of ever-partnered women in Mbeya had experienced physical violence by an intimate partner at some point in their lives [2]. The 2010 Tanzania Demographic and Health Survey (TDHS) found that 39% of ever-partnered women had experienced physical violence by their current/most recent partner in their lifetime [35]. Despite the increasing numbers of studies exploring factors associated with partner violence against women in Tanzania [36–38], few have explored abused women’s help-seeking behaviour. To date, the most in-depth study analysed qualitative data from DSM, Mbeya and Iringa Regions gathered in 2009 [32]. From participatory focus group discussion and key informant interviews, the study explored patterns of help-seeking and highlighted how gendered norms around the acceptability of violence, fear of shame, stigma and/or an escalation of violence, and a lack of trust in the response system meant abused women tolerated violence and were prevented from seeking help [32].

## Methods

This study used existing cross-sectional household survey data from women aged 15–49 collected between November 2001 and March 2002. In both sites a multi-stage sampling approach was used with each location being divided into districts, and then clusters within selected districts. Households were randomly selected within each cluster. The age and initials of all females in each household were recorded, and one respondent aged 15–49 per household was interviewed. In situations where there was more than one eligible respondent, one woman was randomly selected. A total of 1820 individual interviews were completed in DSM and 1450 in Mbeya, with an individual response rate of over 96% in both settings. Further details on the study design are explained elsewhere [6].

Respondents were asked detailed questions about themselves and their community; their general and reproductive health; their children; their attitudes towards gender roles and financial autonomy; their current or most recent partner and their experience of violence; injuries they may have sustained because of violence; and their impact and coping mechanisms.^2^ ^2^Women who experienced sexual violence only (122 in DSM and 116 in Mbeya) were not asked about impact and coping mechanisms.


### Physical partner violence

The questionnaire recorded responses for each woman on her experience of six different acts of physical violence by her partner (whether she had ever been slapped or had something thrown at her, pushed, hit with fist or something that could hurt her, kicked or dragged, choked or burnt, or threatened with a knife, gun or other weapon). Of the women who had ever been partnered (1442 in DSM and 1256 in Mbeya), 474 in DSM and 586 in Mbeya had experienced at least one act of physical violence by a male intimate partner in their lifetime [6].^3^ ^3^Sample excludes women who had never been partnered and women who did not respond to questions about partner violence.


In order to distinguish between moderate and severe physical violence, injuries sustained because of violence were also considered. Women who experienced having been slapped and/or pushed and no injury were considered as having experienced moderate physical violence only, and women who experienced either being hit with fist, kicked or dragged, choked or threatened and/or injury because of violence were considered to have experienced severe physical violence.

### Measures of women’s responses to violence

Women who reported having ever experienced physical violence by an intimate partner were asked additional questions about whether they revealed their circumstances to anyone; sought help from anyone; reasons for seeking help (among those who sought help) and reasons for not seeking help (among those who did not seek help); whether they fought back physically; whether they left home even if for one night and if so, the number of times they left home, reasons for leaving home, where they went, how long they stayed away, and reasons for returning (among those who returned).

### Indicators of women’s economic status

Characteristics of women’s economic status included their educational attainment coded as *No schooling, incomplete primary, complete primary,*and *some secondary or more*; whether they earned money; ownership of three different types of capital assets – land, a house or a business – each coded as *doesn’t own; owns jointly;*and *owns independently*; and *w*hether they were able to raise cash in an emergency.

### Analysis strategy

Multivariate logistic regression was used to assess the relationship between the six indicators of women’s economic status and help-seeking and separation (temporary and permanent) from their abusive partners. Each indicator of women’s economic status was modelled separately and adjusted for the women’s age; severity of violence; acceptance of wife beating; and witnessing violence in childhood.^4^ ^4^Acceptance of wife beating was based on the respondents’ opinions that a man has a good reason to hit his wife under at least one of six circumstances: (1) she does not complete her household work; (2) she disobeys him; (3) she refuses to have sexual relations with him; (4) she ask him whether he has other girlfriends; (5) he suspects she is unfaithful; (6) he finds out she has been unfaithful. The sample used in the analysis to model separation from partners were all women who had ever lived with a violent male partner (n = 432 in DSM and n = 570 in Mbeya). All analyses were carried out using Stata version 13.0.

## Results

### Women’s socioeconomic and demographic characteristics

The socioeconomic and demographic characteristics of ever physically abused women in DSM and Mbeya are shown in Table 1. Women’s mean age was similar in both sites at slightly under 31 years. Three-quarters of women in DSM and 84% of women in Mbeya were either married or cohabiting with their partner at the time of interview. While 15.4% of women in DSM were in regular dating relationships, few women in Mbeya (3.6%) were dating. Slightly under 10% in DSM and 12.5% in Mbeya were not partnered at the time of the survey of which, in DSM, almost three-quarters were either divorced or separated, compared to just over one-half of women in Mbeya. In DSM, over one-half (57.1%) are Muslim with the remaining women reporting they are Christian. By contrast, over three-quarters of women in Mbeya are Christian and very few (2.4%) Muslim; the remaining reported they followed either no religion or other religion.

**Table 1.** Women’s socio-demographic characteristics in DSM and Mbeya.

	DSM	Mbeya
	N = 474	N = 586
Age	30.95 *(8.21)*	30.89 *(7.85)*
Partnership status		
Married	50.4	52.4
Cohabiting	24.5	31.6
Dating	15.4	3.6
Divorced/separated/widowed	9.7	12.5
Religion		
None	0.0	14.0
Islam	57.4	2.4
Christian	41.8	76.1
Other	0.8	7.5
Education		
None	11.0	29.2
Incomplete primary	12.2	13.8
Complete primary	58.4	50.9
Some secondary +	18.4	6.1
Earns money	53.2	66.8
Land		
Owns with others	26.8	50.2
Owns by self	15.4	26.5
House		
Owns with others	27.2	61.8
Owns by self	10.8	10.6
Business		
Owns with others	4.0	6.0
Owns by self	14.8	10.1
Raise money in an emergency		
Yes	41.7	53.2
(Dating/not partnered)	25.2	16.1
Household S.E.S		
Low	67.9	91.1
Middle	21.7	7.2
High	10.3	1.7
Parity		
0	12.0	5.3
1–2	43.3	30.4
3–4	27.2	33.3
5 +	17.5	31.1
Attitudes to wife beating	71.2	75.7
Violence in childhood	47.2	59.5

Note: All figures are given in percentages except those for Age which are given as: mean *(S.D)*.

Women in DSM had higher educational attainment compared with women in Mbeya – on average, two more years of schooling. In Mbeya, 43% of women had not completed primary school and only 6% had at least some secondary education compared to less than one-quarter who had not completed primary school and almost one in five women who had at least some secondary schooling in DSM. Women in Mbeya, however, appeared to be more economically endowed than women in DSM. In Mbeya, two-thirds of women earned money and approximately three-quarters owned land or a house either jointly or independently; in DSM the figures were 54% (earns money), 43.5% (land ownership) and 39.4% (house ownership). Although fewer women in DSM earned money, among those who did, proportionately more controlled the income that they earned and few gave all their income to their husband/partner. For example, among married or cohabiting women (to whom the question on control of income was asked) 85% controlled their income in DSM compared to less than two-thirds in Mbeya (data not shown). In both sites, fewer than 20% of women owned a business; and the proportion who owned a business independently was slightly higher in DSM (14.8%) than in Mbeya (10.1%). Just over half of women in Mbeya reported they would be able to raise money in an emergency, and this figure was 41.7% in DSM.

The vast majority of households in Mbeya were classified as low socioeconomic status (SES) compared to two-thirds of households classified as low SES in DSM. Parity was higher in Mbeya than in DSM, with fewer women reporting no children and almost one-third reporting five or more children. The majority of women agreed with at least one reason why it is acceptable for a man to beat his wife (71.2% in DSM and 75.7% in Mbeya); more women in Mbeya reported their mother had been hit by their father (47.2% in DSM and 59.5% in Mbeya).

### Women’s experiences of partner violence

Women’s experiences with violence were similar in both sites (Table 2). The proportion of women who had experienced severe physical violence was 56.1% in DSM and 57.8% in Mbeya, and among those who had experienced severe physical violence, slightly over one-half reported that they had received injuries because of the violence (51.9% in DSM and 51.3% in Mbeya). In both sites, slightly over 30% of women reported that their husband/partner’s violence had affected their physical or mental health – a figure that rises to 44% among women who experienced severe physical violence.

**Table 2.** Women’s experiences of physical partner violence in DSM and Mbeya.

	DSM	Mbeya
	N = 471	N = 583
Severe physical violence	56.1	57.8
Injured because of violence	29.1	29.7
Severe physical*(N =)*	*(264)* 51.9	*(337)* 51.3
Physical/mental health	31.2	30.8
Moderate physical violence*(N =)*	*(206)* 14.6	*(245)* 12.2
Severe physical violence*(N =)*	*(263)* 44.5	*(234)* 44.0

### Women’s responses to partner violence

Virtually the same proportion of women who had ever experienced physical partner violence had disclosed their experiences to someone in both sites (69.6% in DSM and 69.1% in Mbeya) or had sought help (40.8% in DSM and 41.2% in Mbeya) (Table 3). There were differences between the two sites in terms of to whom women disclosed or where they sought help. In DSM, women most commonly disclosed to their own family (50%) or their partner/husband’s family (29.3%); the most common places where help was sought were hospitals (20.3%), religious leader (20.0%) and police (15.2%). In Mbeya, proportionately fewer women, compared to women in DSM, disclosed the violence to members of their own family (34.8%) – though this was still the most common form of disclosure – and proportionately more women disclosed to their friends or neighbours (28.8%) or their local leader (25.4%). Women in Mbeya were also less likely to have sought help from the police (6.5%) or from a health professional (13.7%) and were more likely to have sought help from their local leader (33.3%).

**Table 3.** Women’s responses to physical partner violence in DSM and Mbeya.

	DSM (N = 474)	Mbeya (N = 586)
Ever told anyone	69.6	69.1
*Informal sources*		
Family	50.0	34.3
Husband/partner’s family	29.3	29.4
Friends/neighbours	21.5	28.8
*Institutional sources*		
Police	6.3	3.4
Health worker	4.6	3.6
Local leader	8.4	24.9
Religious leader	3.2	2.9
Ever sought help from any source	40.8	41.2
Police	15.2	6.5
Hospital	20.3	13.7
Legal advice centre/court	5.7	5.3
Social (shelter/woman’s organisation)	4.2	1.4
Local or religious leader	20.0	33.3
Ever fought back	36.3	16.0
*Number of strategies used (told someone, sought help, ever fought back)*		
No strategy	20.5	26.6
One strategy	29.1	30.0
Two strategies	34.0	34.0
All three strategies	16.5	9.4
*Ever lived with a man (N =)*	*439*	*576*
Ever left (at least one night)	39.4	30.7
Mean number of times left	2.42	2.08
Permanent separation	12.5	9.2

In both sites, the vast majority of women who sought help (193 in DSM and 241 in Mbeya) reported that they had sought help either because they could not endure the violence any longer or because they had been injured – 60%, in both sites, reported that they could not endure the violence any more, and almost 25%, in both sites, reported that they had been badly injured. Of those who did not seek help (280 in DSM and 344 in Mbeya), 56% in DSM and 47.7% in Mbeya reported it was because the violence was not serious. Further, in both sites, 44% reported that they did not want to seek help from anyone, and of those who did, the most common source women wanted help from was family members (data not shown).

Women in DSM were more likely to use physical self-defence against their partner than women in Mbeya – 36.3% of women in DSM reported that they had ever fought back compared to only 16% of women in Mbeya.

The majority of women, in both sites, used at least one of the three (fought back, told someone and sought help) response strategies (79.5% in DSM and 73.4% in Mbeya); however, slightly more women in DSM used all three strategies compared to women in Mbeya (16.5% and 9.4%, respectively).

Almost 40% of women in DSM and slightly over 30% of women in Mbeya had ever left their partner (at least temporarily); 12.6% in DSM and 9.2% in Mbeya had left permanently. Of the women who reported that they had ever left their partner, the average number of times a woman had left was 2.42 in DSM and 2.08 in Mbeya. In DSM, there was no significant difference in the mean number of times a woman had left when stratified by temporary or permanent separation (2.25 temporary; 2.79 permanent; *p* = 0.193); however, in Mbeya, women who had permanently separated were significantly more likely to have left more times than women who had left and who later returned (3.06 permanent; 1.66 temporary; *p* = 0.003) (data not shown). Of those who left their partner (173 in DSM and 177 in Mbeya), over 60% in both sites reported they left because they could not endure the violence any more (61.3% in DSM and 66.7% in Mbeya) and one in five women in Mbeya reported they had left because they had been badly injured (data not shown).

Results from the regression analyses on the association between women’s economic status and having ever sought help are shown in Table 4. Adjusting for women’s age, severity of violence, witnessing mother being hit by father and acceptance of wife beating, women who had primary (incomplete or complete) education, compared to no schooling, who independently owned land or a house were significantly more likely to have sought help in DSM – ownership of a house was borderline significant (*p* = 0.057). In Mbeya, earning money and independent ownership of a house or a business were significantly associated with higher odds of help-seeking – earning money and ownership of a house were borderline significant (*p* = 0.092 and *p* = 0.061, respectively).

**Table 4.** Women’s economic status and help-seeking in DSM and Mbeya.

	DSM				Mbeya			
	O.R	95% C.I		*p*-value	O.R	95% C.I		*p*-value
Education *(None)*	1				1			
Incomplete primary	2.59**	1.10	6.08	0.029	1.20	0.67	2.17	0.537
Complete primary	1.81*	0.90	3.62	0.094	0.84	0.54	1.32	0.451
Some secondary +	0.93	0.40	2.16	0.865	0.73	0.31	1.73	0.471
Earns money *(Doesn’t earn money)*	0.73	0.48	1.10	0.134	1.39	0.93	2.07	0.111
Land ownership *(Doesn’t own)*	1				1			
Joint ownership	0.97	0.59	1.60	0.911	0.68	0.43	1.08	0.100
Independent ownership	2.15**	1.17	3.96	0.014	1.33	0.79	2.23	0.286
House ownership *(Doesn’t own)*	1				1			
Joint ownership	0.93	0.56	1.52	0.765	0.73	0.48	1.10	0.134
Independent ownership	1.99*	0.98	4.03	0.057	1.91*	0.97	3.77	0.061
Business ownership *(Doesn’t own)*	1				1			
Joint ownership	0.83	0.29	2.43	0.738	1.25	0.58	2.69	0.574
Independent ownership	0.95	0.53	1.67	0.847	2.13**	1.16	3.93	0.015
Raise money in emergency *(No)*	1				1			
Yes	1.20	0.75	1.93	0.446	1.15	0.76	1.76	0.508
Not currently partnered	1.41	0.82	2.43	0.208	1.41	0.81	2.48	0.226

Notes: Adjusted for women’s age, acceptance of wife beating, severity of violence, and violence in childhood.

*p < 0.1; **p < 0.05.

Table 5 presents the findings from the analyses of women’s economic status and having ever left an abusive partner. No significant relationship between any of the six indicators of women’s economic status and ever leaving in DSM was found – no economic factor was even close to achieving significance. In Mbeya, four of the six economic indicators were associated with women ever leaving. Women who owned land with others had 44% lower odds of having ever left an abusive partner and this was borderline significant (OR = 0.56; *p* = 0.016), and women who owned a house with others had 53% lower odds of having ever left (OR = 0.47; *p* = 0.001). There were borderline significant associations between having incomplete primary schooling and ability to raise money in an emergency with higher odds of ever leaving. Women who had incomplete schooling, compared to no schooling, had 1.70 times higher odds of ever leaving (*p* = 0.079) and women who reported they were able to raise money had 1.47 times higher odds of having ever left an abusive relationship (*p* = 0.094).

**Table 5.** Women’s economic status and ever leaving in DSM and Mbeya.

	DSM				Mbeya			
	O.R	95% C.I		*p*-value	O.R	95% C.I		*p*-value
Education *(None)*	1				1			
Incomplete primary	1.46	0.62	3.41	0.387	1.70*	0.94	3.06	0.079
Complete primary	1.24	0.63	2.47	0.533	0.87	0.55	1.38	0.544
Some secondary +	0.79	0.33	1.89	0.602	1.00	0.41	2.44	1.00
Earns money *(Doesn’t earn money)*	0.91	0.59	1.41	0.681	1.42	0.93	2.17	0.106
Land ownership *(Doesn’t own)*	1				1			
Joint ownership	1.21	0.72	2.04	0.468	0.56**	0.35	0.90	0.016
Independent ownership	1.08	0.58	2.01	0.817	0.75	0.44	1.26	0.277
House ownership *(Doesn’t own)*	1				1			
Joint ownership	1.14	0.68	1.92	0.608	0.47**	0.31	0.72	0.001
Independent ownership	1.16	0.57	2.37	0.688	0.80	0.41	1.55	0.504
Business ownership *(Doesn’t own)*	1				1			
Joint ownership	0.38	0.10	1.46	0.159	0.65	0.28	1.51	0.311
Independent ownership	1.12	0.62	2.00	0.706	1.30	0.71	2.37	0.393
Raise money in emergency *(No)*	1				1			
Yes	1.12	0.68	1.82	0.657	1.47*	0.94	2.32	0.094
Not currently partnered	1.73	0.96	3.12	0.070	2.58	1.43	4.64	0.002

Notes: Adjusted for women’s age, acceptance of wife beating, severity of violence, and violence in childhood.

*p < 0.1; **p < 0.05.

## Discussion

This study sought to understand women’s responses to partner violence in two Tanzanian settings – DSM and Mbeya. Although multiple of forms of partner violence exist, such as sexual violence and emotional and psychological abuse, questions on responses were restricted to women reporting physical partner violence. Despite this limitation, to date, no detailed population-based study on women’s help-seeking behaviour exists from Tanzania. Therefore, this study provides critical evidence on an aspect of partner violence while also urging additional research to understand women’s coping mechanisms associated with other forms of abuse.

Although the two study sites contrast in terms of social and demographic characteristics, this study has found similarities in women’s experiences of abuse. In both sites, many abused women suffer physical violence from a male intimate partner that can be classified as severe, with similar proportions experiencing injuries as a result of the violence and reporting adverse physical and mental health effects.

This study also found similarities in women’s responses to partner violence. The majority of women had told someone, predominantly family or friends, about their experiences of violence – a finding that dispels a common assertion about the extent to which partner violence against women is hidden. The proportion of women seeking help, approximately 40% in both sites, is also higher than that reported in urban and rural Bangladesh and Nicaragua [11,14]. Help-seeking from formal institutions (police, hospital, legal services and social protection e.g. shelters) was, however, very low and particularly so in Mbeya. This has not changed in the 10 years since the WHO study was conducted. The 2010 TDHS found that 44.8% in DSM and 36.8% in Mbeya of (physically or sexually) abused women had sought help and that nationally, help was most commonly sought from family members or religious leaders and was least commonly sought from police, legal assistance, health professionals or social services [35].

That few abused women seek help from formal sources may be a reflection of the limited availability of those support services that exist, particularly in provincial areas. The low level of help-seeking in DSM, where services are likely to be concentrated, however, highlights that additional factors influence women’s help-seeking behaviour. Women in both sites reported that they would like to receive more help, but when asked whom they would like to receive help from, many women stated members of their family rather than police or health centres. These preferences are likely to still exist as the qualitative study in Tanzania by McCleary-Sills and colleagues documented how women seek help from various informal sources first before approaching formal services [32]. Women’s first port of call was the family, at times the partner’s family, where the support given varied from assistance in mediation between the woman and her partner to advice including being told to tolerate the abuse. It is only if the issue remains unresolved that it is then acceptable for women to go to external sources of support such as local or religious leaders [32]. A qualitative study on health care worker perceptions about intimate partner violence, conducted in DSM, highlighted their frustrations at women’s reluctance to disclose abuse despite their being given evidence from the victims’ families or friends. The study concluded women consider abuse to be a shameful domestic matter or that revealing the violence would cause the woman stigma [39].

These findings suggest community-based interventions that encourage community members to challenge existing societal attitudes towards the acceptability of partner violence against women may be valuable. For example, mobilising communities and sensitising community leaders on the role that traditional, and inequitable, gender norms play in perpetuating violence against women may encourage more sensitive and responsive solutions for abused women.

Much has been written in the literature about women separating from their partner when the violence is severe [10,11,14]. This study conforms to this finding and the reasons women gave for seeking help or leaving the relationship were because they ‘could not endure the violence any more’ or because they had received serious injuries. Many women who had never left said this was because they did not consider the violence that they experienced as serious; and underlying this may be the normative acceptance of wife beating under certain circumstances [32,40,41].

The second main objective of this study was to explore the role of women’s economic status on their responses to partner violence. A second limitation from this study to highlight is that the causal relationship between economic factors, with the exception of educational attainment, and responses cannot be determined because of the cross-sectional design of the study. That is, it is not clear whether women’s economic status determines their responses to violence or whether women’s responses shape their decision to acquire work or to own assets. Therefore, the results from the multivariate regression analyses should be interpreted with caution.

This study found no association between the different indicators of women’s economic status and ever leaving an abusive relationship in DSM. In Mbeya, however, having some primary schooling, compared to no schooling, and ability to raise money in an emergency were both associated with increased odds of leaving abusive partners. The non-significant association, in both sites, with women’s employment is consistent with a study in urban India. The study concluded that the importance of paid work in promoting decisions to leave violent men was mitigated by social considerations, and that issues around the control over their income and women’s work being along gendered lines, e.g. low paid and within the informal sector, also inhibited women’s ability to leave [25]. Some of these conclusions were mirrored in findings from a qualitative study conducted among women market traders in DSM and Mbeya. The study documented the expectation among women that they remain with their husbands but that among women who were able to flee severe abuse, it was strong natal support, rather than their employment, that enabled them to escape [41].

Proposed as a key empowerment strategy, increasing women’s property ownership is considered to enhance women’s livelihood options, conferring greater security on them and providing them with somewhere to go and immediate escape options from domestic violence [42]. For example, in a study carried out in Kerala, many women who faced violence and who owned property outside of the marital home were able to leave the situation [43]. In this study, none of the indicators of either joint or independent capital asset ownership – land, house or business – were associated with leaving in DSM; however, joint ownership of land or a house was significantly associated with reduced odds of women leaving in Mbeya – possibly reflecting the ties that bind women in relationships when immovable assets are shared.

Although there were limited associations found between women’s economic status and leaving an abusive partner, women’s economic status appears to have played a role in women’s ability to seek help. In particular, women’s independent ownership of capital assets appeared to confer on them the resources needed to seek help – land and house in DSM and house and business in Mbeya. Earning money also supported women’s ability to seek help in Mbeya. This finding may be due to a combination of factors including the networks that working women establish. However, more importantly, women’s earnings and economic resources may reduce a barrier of costs (both direct costs, e.g. hospital registration, and indirect costs, e.g. transport) that may prohibit women from seeking help [32,39]. A study from Uganda found that the out-of-pocket expenditure related to a single incident of partner violence amounted to $5.00 from a health centre and $10.00 when seeking police intervention – costs that are significant when considering the Gross National Income per capita of $340 [44].

## Conclusion

Since this study was conducted, the Government of Tanzania has increased efforts to respond to gender-based violence in the country. Patterns of help-seeking, however, have remained the same with the majority of women seeking help from family and friends and only seeking help from formal institutions or leaving the partner when the violence has become severe. Efforts, therefore, need to continue to provide and strengthen services that will encourage women to seek the help from formal sources that they need, and to make it more accessible. In addition, community-based interventions that would assist in resolving cases in a more gender-sensitive way should also be considered.

## Paper context

In many countries around the world partner violence against women remains a silent epidemic with many women concealing their experiences of abuse. This study from Tanzania highlights the extent to which abused women, from two contrasting settings, desire assistance and especially so from family and community members. As Tanzania increases its multi-sectoral response efforts, other approaches such as community-based interventions promoting non-tolerant attitudes towards domestic violence could also play an important role in resolving cases.
